# Evolution and genomic organization of muscle microRNAs in fish genomes

**DOI:** 10.1186/s12862-014-0196-x

**Published:** 2014-09-25

**Authors:** Pedro Gabriel Nachtigall, Marcos Correa Dias, Danillo Pinhal

**Affiliations:** Department of Genetics, UNESP - Sao Paulo State University, Institute of Biosciences, Botucatu, SP 18618-970 Brazil; Institute of Health Sciences, UFMT -Federal University of Mato Grosso, Mato Grosso, 78550-000 Brazil

**Keywords:** miRNA, Comparative genomics, Striated muscle, Evolution, Paralogs

## Abstract

**Background:**

MicroRNAs (miRNAs) are small non-coding RNA molecules with an important role upon post-transcriptional regulation. These molecules have been shown essential for several cellular processes in vertebrates, including muscle biology. Many miRNAs were described as exclusively or highly expressed in skeletal and/or cardiac muscle. However, knowledge on the genomic organization and evolution of muscle miRNAs has been unveiled in a reduced number of vertebrates and mostly only reflects their organization in mammals, whereas fish genomes remain largely uncharted. The main goal of this study was to elucidate particular features in the genomic organization and the putative evolutionary history of muscle miRNAs through a genome-wide comparative analysis of cartilaginous and bony fish genomes.

**Results:**

As major outcomes we show that (1) miR-208 was unexpectedly absent in cartilaginous and ray-finned fish genomes whereas it still exist in other vertebrate groups; (2) miR-499 was intergenic in medaka and stickleback conversely to other vertebrates where this miRNA is intronic; (3) the zebrafish genome is the unique harboring two extra paralogous copies of miR-499 and their host gene (Myh7b); (4) a rare deletion event of the intergenic and bicistronic cluster miR-1-1/133a-2 took place only into Tetraodontiformes genomes (pufferfish and spotted green puffer); (5) the zebrafish genome experienced a duplication event of miR-206/-133b; and (6) miR-214 was specifically duplicated in species belonging to superorder Acanthopterygii.

**Conclusions:**

Despite of the aforementioned singularities in fish genomes, large syntenic blocks containing muscle-enriched miRNAs were found to persist, denoting colligated functionality between miRNAs and neighboring genes. Based on the genomic data here obtained, we envisioned a feasible scenario for explaining muscle miRNAs evolution in vertebrates.

**Electronic supplementary material:**

The online version of this article (doi:10.1186/s12862-014-0196-x) contains supplementary material, which is available to authorized users.

## Background

The massive sequencing of eukaryote genomes has lead to an unprecedented discovery of fundamental features of an abundant class of non-coding regulatory elements, the microRNAs (miRNAs). Recognized as the micromanagers of gene expression, miRNAs added a new layer of complexity to the regulation of protein-coding genes, owing to its ability of virtually silence any mRNA in the cell [1].

The occurrence of deeply conserved and species-specific miRNAs in metazoan, argues in favor of a permanent, rapid and uneven evolutionary process of miRNA genes emergence [2]. Notably, episodes of miRNA repertoire acquirement positively correlate to increased animal complexity [3,4]. Actually, several studies revealed that the origin and evolution of miRNA loci resemble that of protein-coding genes, being prompted by classical evolutionary forces, such as duplication, mutation and genetic drift. Such dynamism generated paralogous members producing identical or nearly identical mature sequences [5,6] many of which are clusterized and expressed in a cell-specific or tissue-enriched basis [7,8].

In this sense, a suite of miRNAs, including miR-1, −133a, −133b, −206, −208a, −208b, −214 and −499, were identified as highly enriched or specifically expressed in cardiac and/or skeletal muscle cells of animals (reviewed in [9]). These muscle-enriched miRNAs are known to play essential roles in muscle biology by controlling processes like myogenesis (proliferation and differentiation), regeneration, aging, homeostasis, apoptosis and immune responses [10-12].

In animals, miRNA expression profiles and functions in muscle and other tissues depend on the miRNA genomic context, which ultimately correspond to miRNA location, conservation and organization in the genome [13,14]. Peculiarities such as the physical distance between miRNA genes in chromosomes [15] or miRNA location in comparison to protein coding genes (i.e., intergenic, intronic, exonic, or mirtron) may provide important clues regarding coordinated regulation and function of miRNAs. However, current knowledge on the genomic context of muscle miRNAs has been unveiled in a reduced number of vertebrates, thus preventing more informative large scale comparisons. Indeed, available data mostly reflects features from mammal miRNAs, whereas fish, the largest class of living vertebrates, remain largely uncharted.

Thus, the aim of this study was to elucidate particular features in the genomic organization and the putative evolutionary history of muscle-enriched miRNAs in fish through genome-wide comparative analysis.

## Methods

### Genome mapping database

Information regarding the genome datasets of the nine fish species deeply analyzed in this study (fugu, *Takifugu rubripes*; pufferfish, *Tetraodon nigroviridis*; medaka *Oryzias latipes*; stickleback *Gasterosteus aculeatus*; zebrafish *Danio rerio*, Nile tilapia *Oreochromis niloticus*, spotted gar *Lepisosteus oculatus*, coelacanth *Latmeria chalumnae* and elephant shark *Callorhinchus milii*) was listed on Table 1. Other fish genomes sources (platyfish *Xiphophorus maculatus*, cave fish *Astyanax mexicanus* and Atlantic cod *Gadus morhua*) were not included in the main analysis owing to uncompleted assemblies and annotations available. All fish genomes were downloaded from Ensembl (release 69; http://www.ensembl.org/index.html), except the genome of the elephant shark (downloaded at http://esharkgenome.imcb.a-star.edu.sg/), being subsequently combined to generate a database using BLAST tool [16].

**Table 1:** **Genome data sets used in the present study**

**Species**	**Common name**	**Scaffold or chromosome**	**Genome (Mb)**	**Assembly**
***Danio rerio***	Zebrafish	Chromosome	1505	Zv9, April 2010
***Oryzias latipes***	Medaka	Chromosome	700	HdrR, Oct 2005
***Gasterosteus aculeatus***	Stickleback	Scaffold	446	BROAD S1, Feb 2006
***Tetraodon nigroviridis***	Tetraodon	Chromosome	342	TETRAODON 8.0, Mar 2007
***Takifugu rubripes***	Fugu	Scaffold	393	FUGU 4.0, Jun 2005
***Oreochromis niloticus***	Nile tilapia	Scaffold	815	Orenil1.0, Jan 2011
***Lepisosteus oculatus***	Spotted gar	Chromosome	945	LepOcu1, Jan 2012
***Latimeria chalumnae***	Coelacanth	Scaffold	2860	LatCha1, Sep 2011
***Callorhinchus milii***	Elephant shark	Scaffold	937	Callorhinchus_milii-6.1.3, Jan 2014

Note - the genomes of *Gadus morhua* (cod), *Xiphophorus maculatus* (platyfish) and *Astyanax mexicanus* (cave fish) were not used here due to their uncompleted assemblies and annotations currently available.

Precursor miRNA (pre-miRNA) sequences retrieved from zebrafish genome at miRBase (release 20, June 2013; www.mirbase.org) were used as reference for successive BLAST searches against our compiled fish database. Recovered matches corresponding to pre-miRNAs of all nine fish species were manually annotated and aligned in order to verify interspecies arrangement and composition. Moreover, several other vertebrate pre-miRNA sequences from amphibians (*Xenopus tropicalis)*, reptiles (*Anolis carolinensis*), birds (*Taeniopygia guttata*) and mammals (*Homo sapiens*, *Mus musculus*, *Monodelphis domestica* and *Ornithorhynchus anatinus*) were retrieved from miRBase and Ensembl, aligned with MUSCLE algorithm [17] using default parameters and compared to orthologs from fish species.

### Genomic context comparative analysis

Comparisons on the physical distance between miRNAs in a same cluster or their exact localization were performed in order to evaluate the changeability in the arrangement of muscle miRNAs in the genome of fishes. Additionally, to better understand the evolutionary dynamics of miR-1, −133, −206, −208, −214 and −499, we performed an analysis of synteny of fish chromosomal segments harboring these miRNA genes. The synteny analysis was conducted in the browser Genomicus (release 69.01; http://www.dyogen.ens.fr/genomicus-69.01/cgi-bin/search.pl) using reference genes annotated in the Nile tilapia genome. Since Genomicus only accept a protein-coding gene name as input data, we designed two workflows based on the genomic organization (i.e., intronic or intergenic) of each muscle miRNA evaluated. For intronic miRNAs the host gene (protein encoding gene) was used as reference. For intergenic miRNAs the closer downstream protein encoding genes (retrieved by the position of miRNA at Ensembl) were used as reference. Only nine protein-coding genes located up and downstream were considered for generating the synteny analysis data. All results generated on Genomicus were manually checked by BLAST searches against fish genomes evaluated. For the elephant shark whose genome was not available at Ensembl database, CDS sequences of the miRNA neighbor genes were retrieved from the Nile tilapia genome and BLAST searched against the elephant shark genome to determine the location of these genes.

### Secondary structure prediction

The secondary structure of all muscle miRNAs were retrieved from the consensus sequence acquired from the alignment of precursor sequences formerly constructed. These analyses were carried out on RNAfold algorithm [18] using default parameters.

Furthermore, we performed an analysis of secondary structure of precursor sequences of miR-214 and miR-214 paralog copy obtained from each fish species in order to verify the paralog compliance to the hairpin structure required by the miRNA biogenesis model.

### Phylogenetic tree

Phylogenetic relationships were constructed based on the 80 bp alignment of precursor sequences of miR-214 from diverse vertebrate species (retrieved from miRBase and Ensembl) as well as miR-214 paralog sequences from fish species, recovered by BLAST, as previously mentioned. Then a maximum likelihood tree was generated on MEGA 5 [19] by using Tamura-Nei model with bootstrap of 1000 replications. Only nodes occurring in over 50% of trees were assumed to be significant. The final tree was adjusted on FigTree v1.3.1 [20].

### Target prediction

Predictions searching for mRNA targets shared by miR-208 and miR-499 were carried out using the online tool TargetScan (http://www.targetscan.org, release 6.2; [21]). Comparisons of shared targets were checked by name in the Gene Ontology database [22].

## Results and discussion

### Precursor and mature miRNA features

Overall, muscle miRNAs genes were highly conserved among species of vertebrates investigated. Interspecific comparisons of fish pre-miRNAs sequences to counterparts from other seven representatives of all living groups of vertebrates (see Additional file 1 for more details) revealed a similarity of roughly 85% for each muscle pre-miRNA (Table 2). Indeed, the comparison of predicted secondary structures of orthologous confirmed that all of them shape into a hairpin structure compliant to the miRNA biogenesis model [1,23]. In the comparison of mature sequences, the degree of similarity among orthologous was even higher, ranging from ~95% to perfectly matched sequences (Table 2). Such findings suggest that muscle miRNAs examined have been under strong selective constraint throughout the evolution of vertebrates, thereby reflecting an acquirement of primordial functions on muscle biology. In fact once a miRNA evolves and acquires a function in a strictly tissue-specific context, it is rarely lost [24,25]. Based on the comparison of here acquired to available data (see Additional file 2), it is possible to infer that all muscle miRNAs investigated are ancient and derived from an early gnathostome ancestor.

**Table 2:** **Comparative analysis of muscle-enriched miRNAs sequences of 16 species representatives of the main vertebrate groups**

**miRNA **	**Sequence length (bp)**	**Mature sequence identity (%)**	**Stem-loop sequence identity (%)**
**miR-1**	71	97.1	81.0
**miR-133a**	73	97.3	91.8
**miR-133b**	76	98.8	86.8
**miR-206**	70	100	83.6
**miR-214**	91	94.3	81.0
**miR-499**	84	95.9	77.8

Burst and expansion of the miRNA repertoire took place at the base of the bilaterian lineage [3-5] and later at the base of vertebrate lineage [3,26]. However it is essential to have in mind that the evolution of miRNAs is an ongoing process and novel miRNAs are emerging [27]. The homologs of ancient miRNAs that emerged more recently by duplication and are still not under functional constraint, may either evolve fast, potentially originating other miRNAs, or be conserved, as verified for the miR-214 paralog identified in the present study (below discussed).

Typically, more ancient miRNAs undergo fewer changes than miRNAs that have emerged more recently, implying that the sequences of young miRNAs have higher evolutionary rates than old miRNAs. This can be attested through an examination of all segments of the hairpin, comprising the mature sequences (5p or 3p) and seed regions. For instance miR-1 and miR-133, whose origin remounts to a common ancestor of Protostomia and Deuterostomia [25], were amongst the most conserved miRNAs (Table 2). By contrast, miRNA clusters such as miR-310 ~ 313 from fruit flies and beetles [28], and miR-290 ~ 295 from mouse embryonic stem cells [29], were found to be young fast-evolving miRNA families. Another fast-evolving miRNA family was recently found in primate, but not in rodent. This emergent primate-specific miRNAs became integrated into ancient gene circuitry improving post-transcriptional control [30].

Thereby we envision that events of miRNAs acquisition and loss might have occurred in basal organisms in response to changes in morphological and physiological traits alongside evolution of vertebrates, as for instance challenges emerged during transition from aquatic to terrestrial ambient. Therefore, the origins of muscle-enriched miRNAs can be related to an acquisition of new physiological and ontogenetic patterns by the gene expression regulatory machinery, which reflects in their constrained evolution [2,31].

### Genomic context of muscle miRNAs

#### Muscle miRNAs genomic organization

Muscle miRNAs have shown remarkable synteny as well as frequent singularities in their organization into fish genomes. Pre-miRNA sequences detected were located into distinct chromosomes (or scaffolds) for each of the six fish species assessed (Table 3).

**Table 3:** **Physical location of muscle-enriched miRNAs in fish genomes**

**miRNA**	**Zebrafish**	**Medaka**	**Stickleback**	**Tetraodon**	**Fugu**	**Nile tilapia**	**Spotted gar**	**Coelacanth**	**Elephant shark**
**miR-1-1/-133a-2**	Chr23	Chr7	S_6	-	-	S_80	ChrLG18	S_6	S_6
									S_121
**miR-1-2/-133a-1**	S_3540	Chr17	S_15	Chr15_r	S_68	S_1	ChrLG9	S_15	S_89
**miR-206/-133b**	Chr20	Chr24	S_21	Chr14	S_72	S_183	ChrLG1	S_21	-
	Chr17								
**miR-208a**	-	-	-	-	-	-	-	S_15	-
**miR-208b**	-	-	-	-	-	-	-	-	-
**miR-214**	Chr20	Chr4	S_138	Chr1	S_13	S_7	S_15	ChrLG10	S_41
miR-214-par*	-	Chr17	S_15	Chr15	S_367	S_131	-	-	-
**miR-499**	Chr11	Chr5	S_18	Chr11	S_79	S_19	S_18	ChrLG18	-
	Chr23								
	Chr23								

Note - “Chr” means Chromosome; “S_” means Scaffold; “-” means absence of gene; “LG” means Linkage Group; *Paralog copy detected in this work.

Looking at miRNAs location we found that miR-1-1/133a-2, miR-1-2/133a-1 and miR-206/133b form bicistronic clusters in fish, exactly as in the genome of mammals [32,33]. We have found a relative conservation of miR-1-2/-133a-1 within its respective intron, once their locations were unchanged for the majority of species. Exceptions were found for spotted gar and elephant shark that presented the miR-1-2/-133a-1 at the same intronic position detected in horse (intron 11). Precursor sequences of clustered miRNAs above mentioned also kept a constant between-genes spacing (see Additional file 3 for more details). On the other hand, the intergenic miR-1-1/-133a-2 genes varied in location, being more sparsely distributed in the genomes of zebrafish, medaka, Nile tilapia and coelacanth (~37.4 Kb, ~22.2 Kb, ~17 Kb, and ~40 Kb long, respectively) than did their homologs in birds and mammals (~10 Kb long) or in stickleback, spotted gar and elephant shark which followed the same arrangement of reptiles and amphibians (~8.8 Kb, ~12.2 Kb and ~9.3 Kb long, respectively).

We found miR-499 inside heavy-chain myosin gene Myh7b gene (also named as Myh14) as previously described for mammals [10] even for the extra copies detected in zebrafish. Our analysis revealed that miR-499 does not have an intronic position conserved in vertebrate species. However, data analysis referred to the expression pattern of this miRNA indicates that the co-transcription within their host gene was conserved as well as the uncoupled transcription from its host gene detected in mouse [34].

Furthermore, miR-214 is clusterized (with miR-199) and intronic of Dnm3 gene as reported in mammals [35]. However neither the widely distributed nor the paralog copy have shown a conserved intronic localization among vertebrates, indicating singularities of miR-214 organization in the genomes analyzed.

#### Muscle miRNAs synteny

**Figure 1:**
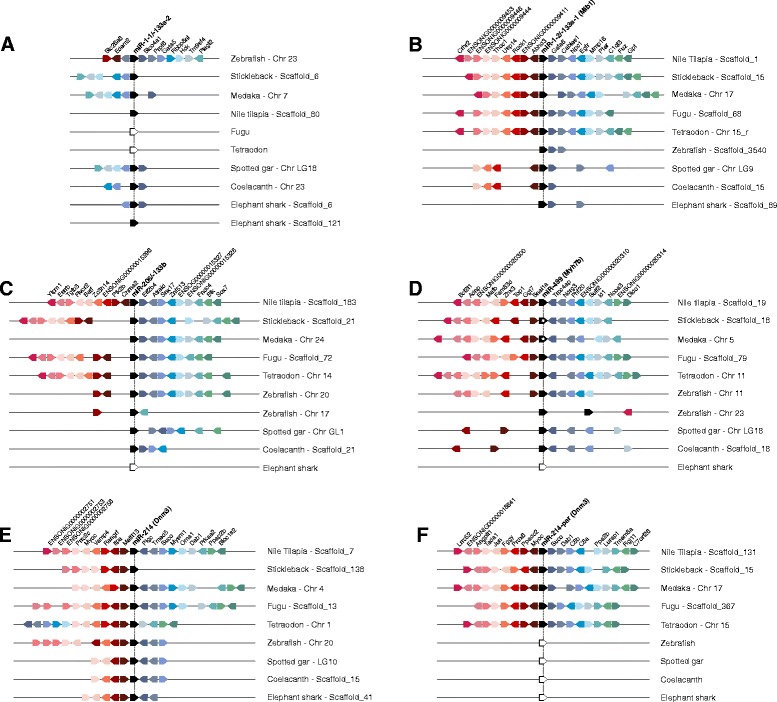
**Chromosomal segments showing the conserved syntenic blocks containing muscle miRNAs in fishes.** The genes are represented by colored pentagons and its name is indicated on top. Color pentagons indicate the same gene in different species (right) and its respective genomic position in relation to several other genes. White pentagons indicate absence of the gene in genome. The pentagon’s direction indicates the gene direction (sense or anti-sense) compared to the reference gene. The empty spaces indicate a region with other genes or repetitive elements. **(A)** miR-1-1/-133a2, in this case we used zebrafish as reference since none syntenic regions were encountered in this cluster within Nile tilapia genome; **(B)** miR-1-2/-133a-1 and its host gene Mib1; **(C)** miR-206/-133b; **(D)** miR-499 and its host gene Myh7b, in this case we used pentagons with white circles to indicate non-intronic miRNA; **(E)** miR-214 and its host gene Dnm3; **(F)** miR-214-par and its host gene Dnm3.

The analysis of muscle-enriched miRNAs gene-neighborhood revealed most genes in high synteny and colinearity within ray-finned fish genomes, with exception made to genes surrounding miR-1-1/-133a-2. The vicinity of this cluster presented none synteny within Nile tilapia genome and low synteny among other fish species analyzed, as just medaka, stickleback and spotted gar had an equivalent structural distribution for a few coding genes (Gata5, Rbbp8nl, Hck, Tm9sf4 and Plagl2), by using zebrafish as reference (Figure 1A). In fact, by extending this comparative analysis to encompass other vertebrate species, synteny was lowered to a single gene (Gata5) as previously demonstrated [36]. Interestingly, elephant shark genome has a duplicated miR-1-1/-133a-2 cluster but only one of the copies maintains the Gata5 gene syntenic to other vertebrate species. Furthermore, we detected that Cables2 gene is located on the same chromosome of miR-1-1/-133a-2 in zebrafish, medaka and stickleback genome but with a larger distance than previously reported [37], having more than nine genes between Cables2 gene and the miR-1-1/-133a-2 cluster (that is why the Cables2 genes are not shown in Figure 1A). We further discovered that the cluster miR-1-1/133a-2 is absent in tetraodon and fugu genomes. In other words, the bicistronic cluster miR-1-1/-133a-2 has an unpredictable arrangement on chromosomes of fish species, thus indicating to be under relaxed evolutionary constraint.

Conversely, conserved blocks of coding genes surrounded the gene-neighborhood of the paralog miR-1-2/-133a-1 cluster (intronic of Mib1 gene) in ray-finned fish genomes (Figure 1B). Our analysis revealed a larger syntenic region among fish species than formerly [36]. Our data showed that Gata6 and Cables1 genes are highly conserved in fish genomes, but when comparing teleost fishes we found a gene block conserved within these genomes composed by genes Abhd3, Rock1, Usp14 and Thoc1. In zebrafish, however, miR-1-2/-133a-1 cluster presents a few conserved protein-coding flanking genes (Gata6 and Cables1), perhaps because it is situated on the *scaffold_3540* that has a limited length of 28,134 bp and has not yet been anchored into zebrafish chromosomes.

The miR-206/-133b cluster presented higher synteny at downstream than at upstream genes (Figure 1C). For instance, medaka, spotted gar and coelacanth upstream region is not syntenic to stickleback, fugu, tetraodon and Nile tilapia, which, in turn, have many common genes in colinearity. For this cluster higher syntenic levels among fish genomes were found for genes Eif2b4, Atraid and Snx17. Zebrafish was the unique species carrying two miR-206/-133b clusters, each at chromosomes 17 and 20. Possibly the cluster at chromosome 17 stand for the young extra copy, since its gene-neighborhood has shown very low synteny levels (only Ptk2b and Fndc4 genes), which indicates a duplication event conjugated within these blocks.

Synteny was also common to coding genes nearby miR-499 orthologs, even in the neighboring of the non-intronic miR-499 of medaka and stickleback genomes (Figure 1D). In this study we recovered 11 syntenic coding genes among Teleostei fishes, whereas Holostei (spotted gar) and Dipnomorpha (coelacanth) showed six genes in sinteny, and none of these genes were synteny to their orthologs in other vertebrate groups. In fact, a unique exception is the Trpc4ap gene that was earlier related as conserved near miR-499 and Myh7b host gene from fish to mammals [37]. Moreover, the two extra copies of miR-499 and Myh7b located at Chr_23 on zebrafish genome displayed a non-syntenic arrangement, since we barely detected a single gene (Bcl2l1) with correspondence at the syntenic block of the miR-499 orthologs shared by the majority of vertebrates.

In general, miR-214 orthologs have been organized as syntenic blocks in fishes although particular features were also uncovered. For instance, the tetraodon genome experienced a rearrangement in the miR-214 neighbor genes Pigc, Tmed5, Suco, Mysm1 and Oma1 moved from downstream to an upstream segment, likely by a translocation episode (Figure 1E). Interestingly, the stickleback genome does not share any genes on its downstream region with other fishes. It is probably caused by the proximity of miR-214 and its host gene to the 3’end of the scaffold sequence they are located. In the case of miR-214-par, it was embedded within a conserved syntenic block on all studied species (Figure 1F).

#### Absence of miR-208 in cartilaginous and ray-finned fish genomes

Unexpectedly, the genome mapping of muscle miRNAs revealed that cartilaginous and ray-finned fish genomes do not retain the miR-208 gene (Table 3), whereas it exists as single-copy in lobe-finned fishes. Thus, to better trace miR-208 evolution, a more exhaustive search on further three fish (platyfish, cave-fish and Atlantic cod) and 50 other vertebrate genomes (41 mammals, five birds, two reptiles, one amphibian and one agnatha; available at Ensembl database) was performed. This survey reinforced that miR-208 gene is absent in cartilaginous and ray-finned fishes and demonstrated that it exists as single-copy not only in lobe-finned fishes, but also in all amphibians, reptiles and birds so far fully sequenced. Additionally, a second paralog copy was detected to all mammals investigated.

Examining the two miR-208 copies recovered in mammals, the paralogs miR-208a and miR-208b, we detected that both were intronic of Myh6 and Myh7, respectively, in agreement to a previous report in mouse and human [38]. We then looked at miR-208 host genes Myh6 and Myh7 and noticed that Myh7 was actually absent into all fish genomes, whereas Myh6 persists but missing the intronic miR-208, except for coelacanth that retain the miR-208 intronic to Myh6. Once two copies of miR-208 intronic of Myh6 (Ensembl: ENSPMAG00000002428 and ENSPMAG00000004862) were reported in the basal Agnatha *Petromyzon marinus*, our findings strongly suggest that miR-208 have been secondarily lost in the cartilaginous and ray-finned fish lineage.

**Figure 2:**
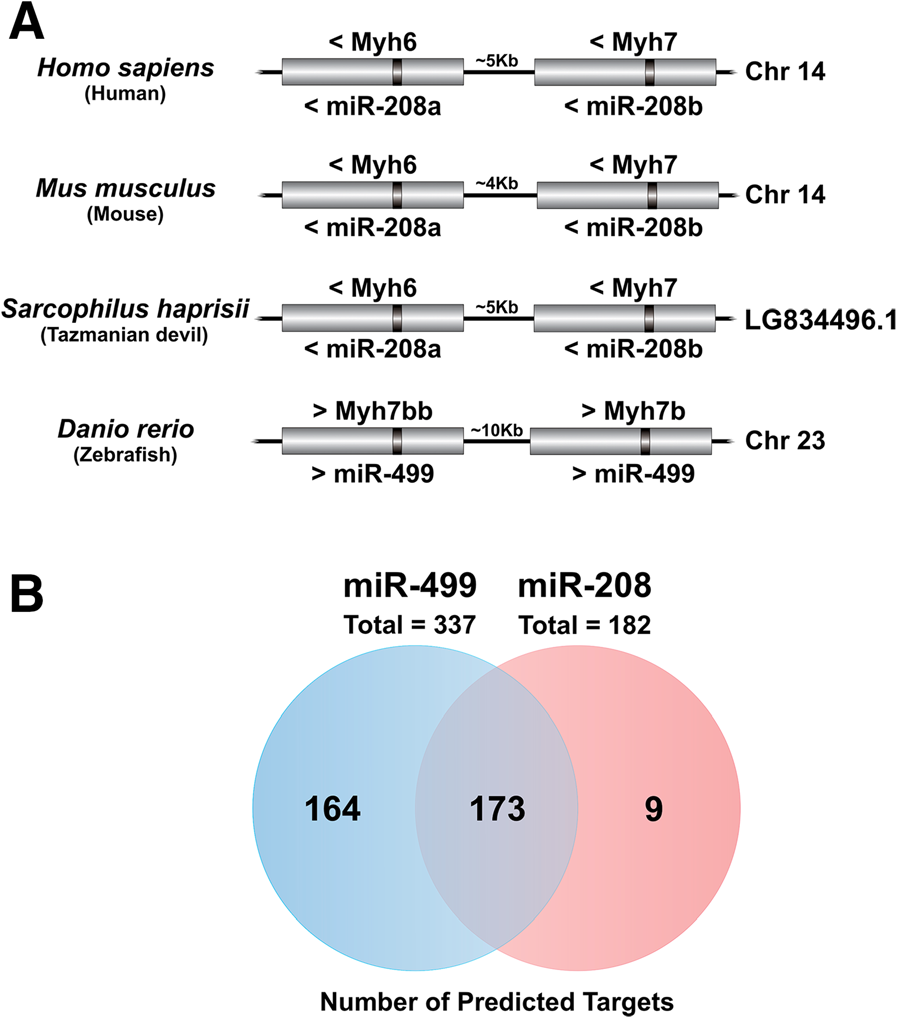
**Genome organization and target prediction analysis of miR-208 and miR-499. (A)** Schematic representation of the genomic organization of miR-208a and miR-208b and their host genes in mammals. In fish, a similar pattern of genomic organization was observed on the doubled additional copies of miR-499 and its host gene detected on zebrafish. “Chr” means Chromosome and “LG” means Linkage Group. **(B)** Number of predicted targets shared by miR-208 and miR-499.

In mammals, miR-208 is required for the expression of miR-499, which is intronic of the Myh7b gene [38]. In the zebrafish genome, which lost miR-208, three extant copies of miR-499 intronic to Myh7b have been previously described [33]. So, we further examined the miR-499 paralogs and find out that two of them are closely located (~10 kb) in the chromosome 23. Curiously, a similar arrangement was detected in mammals for the two intronic miR-208 paralogs and their myosin host genes (Figure 2A). Otherwise, the majority of vertebrate species presented a single intronic copy of miR-499 within Myh7b, physically unlinked to miR-208 paralogs and their Myh6 and Myh7 host genes. Moreover, we found that tetraodon, fugu and Nile tilapia genomes carry a second non-sintenic copy of Myh7b that does not harbor the intronic miR-499, whereas medaka and stickleback have a single non-intronic miR-499 gene.

Together our data regarding miR-499 genomic organization in fish genomes corroborates recent findings from Bhuiyan et al. [37], which have found medaka miR-499 expression patterns are comparable to those reported in zebrafish [26] despite the uncoupling condition of these genes in medaka genome. However, data about the expression pattern of Myh7b in fish genomes carrying non-intronic miR-499 has not been formerly reported. The miR-208 is considered essential for the differentiation and maintenance of slow-twitch muscle fiber type [10,38] as well as for heart development and health [39]. Our data suggests that the lack of miR-208 might be counterbalanced by miR-499 once they have the same seed sequence and consequently share the majority (~95%) of predicted targets (Figure 2B). This assertion was further supported by a comparison of the frequency of shared targets for any two miRNAs chosen at random to the frequency of targets shared by miR-208 and miR-499. Such analysis undoubtedly demonstrates that miR-208 and miR-499 share larger portion of common targets than expected by chance (Table S1, Additional file 4).

We gathered additional evidence of exchange of function between miR-208 and miR-499 after mining published miRNA sequencing datasets of several vertebrates [40-43]. From this review we noticed that miR-208 was highly and weakly expressed in cardiac muscle of mouse and lamprey, respectively, whilst miR-499 was highly expressed in zebrafish and zebra finch cardiac muscle (see Table S2 in Additional file 4 for more details).

Hence, reconstruction of miR-208 evolutionary history remounts to a birth event of miR-208 in an ancient vertebrate lineage with its secondary loss on cartilaginous and ray-finned fish lineages and maintenance in the lineage that raises sarcopterygii and tetrapod lineages followed by a duplication event restricted to mammal genomes.

#### miR-214 duplication

In the comparative screening of the genomic context of muscle miRNAs in vertebrates, we found that all non-fish species carry a single copy of miR-214 and its host gene Dnm3. In fish, however, a variable by-species pattern exists. For instance zebrafish, common carp, Atlantic cod, coelacanth, spotted gar, cave fish and elephant shark follow the single copy pattern of non-fish species whilst platyfish, stickleback, medaka, fugu, tetraodon and Nile tilapia possess two copies of miR-214 and Dnm3. This extra miRNA copy turns out to be a novel paralog of miR-214, henceforth called miR-214-par.

**Figure 3:**
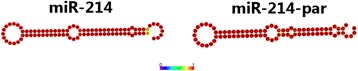
**Secondary structures of miR-214 and miR-214-par.** The sequences of Nile tilapia were used to demonstrate the hairpin structure of the paralogs. Rainbow colored bar indicates the ligation probability between bases (0 = lowest probability; 1 = highest probability).

We then looked at the miR-214-par precursor sequences and verified that they were distinct to precursors of the canonical miR-214 by twelve to fifteen nucleotide substitutions in 90 bp nucleotide sequences. However, the predicted secondary structure of miR-214-par precursors mounts into a hairpin conformation consistent with the miRNA biogenesis model (reviewed in [5]), therefore denoting functionality to the paralog copy detected (Figure 3).

**Figure 4:**
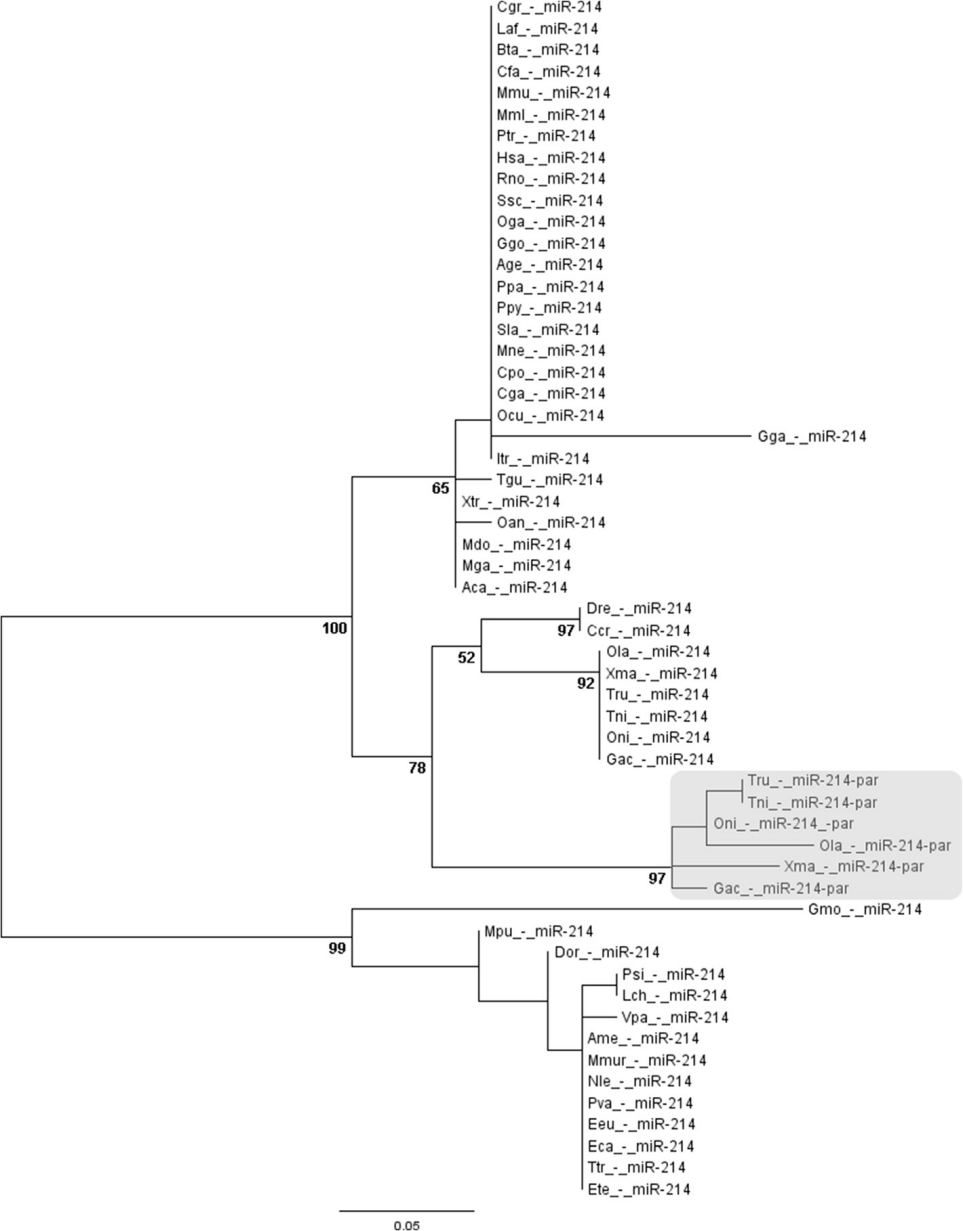
**Phylogenetic tree of miR-214 in vertebrates.** Highlighted square represents the divergent clade of miR-214-par. Aca – lizard; Age – spider monkey; Ame – panda; Bta – cow; Ccr – common carp; Cga - marmoset; Cgr–chinesehamster; Cpo – Guinea pig; Dor – kangaroo rat; Dre - zebrafish; Eca – horse; Eeu – hedgehog; Ete – tenrec; Gac – stickleback; Gga – chicken; Ggo - gorilla; Gmo – cod; Hsa – human; Laf - elephant; Lch - celacanth; Mdo – opossum; Mga – turkey; Mml – rhesus; Mmu – mouse; Mne - macaque; Mmur - lemur; Mpu - ferret; Nle - gibbon; Oan – platypus; Ocu - rabbit; Oga - bushbaby; Ola - medaka; Oni – Nile tilapia; Ppa - bonobo; Ppy - orangutan; Psi – chinese turtle; Ptr – chimpanzee; Pva - megabat; Rno - rat; Sla - marmoset; Tgu – zebra finch; Tni – tetraodon; Tru – fugu; Ttr – dolphin; Vpa - alpaca; Xma – platyfish; Xtr – frog.

In the phylogenetic tree, all miR-214-par branched out together, founding an isolated clade from the canonical miR-214 (Figure 4). Paralogs were closely related to their canonical counterparts, who formed a branch exclusively composed by fish species, further supporting the origin of paralogs restricted to the fish lineage. Then, we can infer that miR-214-par derived from miR-214 by duplication and divergence, being secondarily lost in a few fish lineages. Such findings are in agreement to an expected dynamic of a particular miRNA gene evolving independently into different species [44].

Our analysis suggests that the duplication event of miR-214 was restricted to medaka, stickleback, tetraodon, fugu, Nile tilapia and platyfish. These species belong to the Acanthopterygii superorder. On the other hand, zebrafish, common carp and cave fish belong to Ostariophysi superorder, Atlantic cod belongs to Paracanthopterygii superorder, coelacanth belongs to Sarcopterygii class and elephant shark belongs to Chondrichthyes class. Thereby, it indicates two possibly scenarios where (1) the duplication event occurred in a bony fish ancestor, and miR-214-par was lost in diverse lineages but kept in superorder Acanthopterygii; or (2) the duplication event was specific to superorder Acanthopterygii. The second scenario is more parsimonious due to loss of functional miRNAs is extremely rare [45].

**Figure 5:**
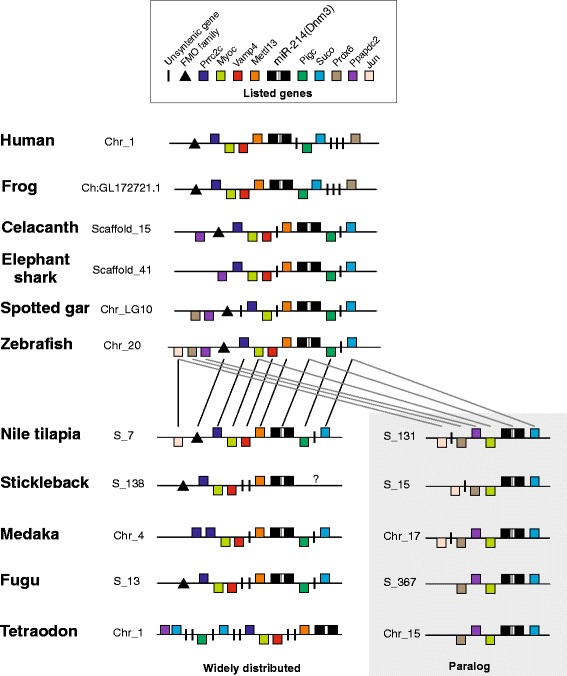
**Schematic representation of the genomic organization and synteny analysis of miR-214 and its paralog detected on fish genomes.** Orthologous genes shared by the widely distributed copy are connected by black lines. Orthologous genes shared by the paralog copy are connected by gray lines. The interrogation dot on 3’ region of stickleback indicates no detection of any gene once miR-214 and its host gene were detected on the region of the scaffold that are not anchored with other scaffold yet. Genes represented above the midline are in sense strands, whereas those represented below are in antisense strands. “Chr_” means chromosome and “S_” means scaffold.

Moreover, the synteny analysis reveals that there is a block of seven genes (Mettl13, Vamp4, Myoc, Prrc2c, Pigc, Suco and Prdx6) highly conserved between human, frog, zebrafish and other fish species. Amid these genes, only Suco and Myoc were duplicated along with miR-214-par, while Mettl13, Vamp4, Prrc2c and Pigc were maintained neighboring the canonical miR-214 widely distributed in vertebrates. By contrast, only Prdx6 gene was retained near of miR-214-par. Furthermore, in fish species we observed that genes nearby miR-214-par were highly syntenic, excepting the Jun gene that was solely retained in Nile tilapia, medaka and stickleback genomes (Figure 5). Conversely, the miR-214-par and miR-214 have shown poor synteny for their respective surrounding protein coding genes. For instance Myoc, Suco, Ppapdc2 genes are maintained in synteny into both miR-214 variants, contrasting to the majority of remaining protein coding genes which have evolved distinctively after duplication. Interestingly, the Prdx6 gene that is located near to miR-214-par was exclusively held by those fish species carrying this miRNA paralog copy.

Therefore, we suggest that the intronic miR-214-par emerged by the duplication of its host gene Dnm3, followed by the transposition of both genes to another chromosome or scaffold. However, extra bioinformatics analyses including an analysis of miRNAs from additional taxa, will be insightful to better endorse such hypothesis.

All the results presented here confirm the miR-214-par as a new paralog of miR-214 and highlight a duplication event restricted to superorder Acanthopterygii, thus fostering the need for a new nomenclature to these miRNA genes (i.e. miR-214-1 and miR-214-2). Besides such miRNA gene duplication, we have identified several protein-coding genes also duplicated, retained in the vicinity. This finding demonstrates an evolutionary constraint as reflex of putative functional relationship.

### Evolutionary scenario of muscle miRNAs in vertebrates

**Figure 6:**
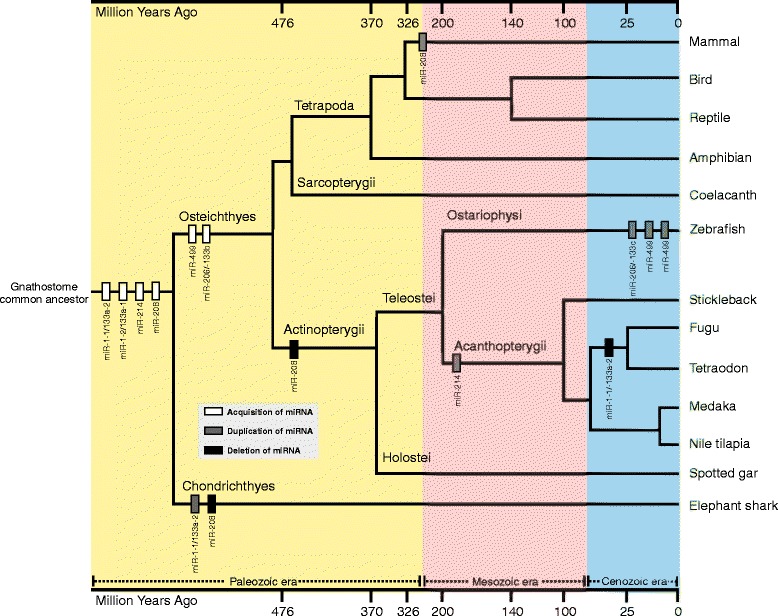
**Putative evolutionary scenario of muscle miRNAs in vertebrates.** The blue, pink and yellow areas represent the Cenozoic, Mesozoic and Paleozoic eras, respectively. The gray square contains the legend for acquisition, duplication and deletion events. The bars on top and bottom (unscaled) represent the timescale in million years.

New data generated for Nile tilapia coupled with available data on several species allows us to propose a general evolutionary scenario for muscle-enriched miRNAs in vertebrates (Figure 6).

In this hypothetical scenario a birth event of the clusterized and intronic miRNA miR-1-2/-133a-1 took place on a common Chordata ancestor [46]. Subsequently, this cluster generated the intergenic cluster miR-1-1/-133a-2 by duplication and rearrangement events. Another duplication of miR-1/-133a cluster must have occurred on a gnathostome ancestor, before the split of Chondrichthyes, Actinopterygii, Sarcopterygii and Tetrapoda lineages. Then, this extra copy of the miR-1/-133a cluster probably acquired a function and was maintained in Chondrichthyes, whereas it was converted into a novel miR-206/-133b cluster retained in the genome of the remaining vertebrate groups over evolution [36]. We also can propose that the miR-206/-133 cluster was just recently duplicated in zebrafish, because extra copies of this locus were not detected in any other vertebrate genome. Furthermore, our survey suggests that a recent event of rare loss of the functional bicistronic miR-1-1/-133a-2 took place into a common ancestor of Tetraodontiformes because both *Fugu* and *Tetraodon* species do not carry this miRNA cluster in their genomes.

Therefore, we can infer that the birth event that doubled miR-208 took place in a common vertebrate ancestor and was followed by a conversion of one locus of miR-208 into miR-499 in a bony fish common ancestor. This pattern was maintained in Sarcopterygii and Tetrapoda, but not in cartilaginous and ray-finned fish lineages that underwent the loss of the miR-208 but not of its host gene. Moreover, we have found the miR-208 distributed in all 41 mammals examined, implying that the miR-208 and its host gene were duplicated on a mammal common ancestor only. Such conclusion is strengthened by the absence of miR-208 in cartilaginous and ray-finned fishes, as well as by the existence of a unique miR-208 copy in Sarcopterygii, birds, reptiles and amphibians.

Regarding the miR-499, all acquired data suggests that this myomiR emerged and was retained under the same organizational pattern in Actinopterygii and Sarcopterygii. However, there are two peculiarities that might have happened for this miRNA: (1) a recent double duplication of miR-499 and its host gene in zebrafish genome, since no similarities of this pattern were detected in other vertebrate genomes; and (2) the conversion from intronic to intergenic miR-499 in medaka and stickleback genomes, an event that might have occurred independently, as recently described for unrelated species Atlantic cod and platyfish [37].

For the miR-214, we propose a gene-birth in a gnathostome common ancestor, because our deep genome search did not recover this miRNA neither in lamprey (agnatha) nor in any other basal chordate. Additionally the highly conserved sequence and organizational pattern within all gnathostome species indicates that miR-214 would have experienced similar evolutionary pressures that kept it under strong selective constraint. Moreover, a duplication event restricted to a common ancestor of Acanthopterygii would explain the miR-214 paralogs in all members investigated from this superorder. Overall, our analysis supports a scenario where the origin of miR-214 in vertebrate genomes took place during Paleozoic and extant paralogs came out after a gene duplication event restricted to an Acanthopterygii ancestor during Mesozoic.

## Conclusions

The high dynamism inherent to fish genomes helps to explain the variable pattern in the distribution of miRNA genes but fails to explain the high synteny observed so far. In this work, we thoroughly studied the genomic organization and discussed the evolutionary dynamics and diverse remarkable singularities of muscle miRNAs in fishes. We also show that, in spite of a few peculiarities and differences between species, some of the chromosomal regions that contain the muscle miRNAs had high synteny levels, denoting colligated functionality. Moreover, we conclude that most of events of muscle-specific miRNAs acquisition and diversification occurred on a gnathostome common ancestor, whereas singular events of miRNA duplication and loss were randomly assigned to distinct species of the divergent animal groups studied. Collectively, these insights about the miRNA genomic context shed new light on the evolutionary history of such key modulators of the muscle biology in vertebrates.

## Additional files

Additional file 1:
**Multiple alignments of precursor sequences of the muscle-enriched miRNAs between vertebrate species and the secondary structure of consensus sequence.** Light gray bar indicates mature sequence; Black gray bar indicates seed sequence. Additional file 2: Table S1. Novel and formerly published data on muscle miRNAs in vertebrate genomes. Additional file 3: Table S1. Structural characteristics of muscle miRNAs in fish genomes. Additional file 4: Table S1. Predicted target genes shared between miR-208 and other miRNAs (including muscle and non-muscle miRNAs). The shared genes are highlighted in light blue. **Table S2**. Read counts for the datasets compared to verify the expression of miR-208 and miR-499 in vertebrate hearts. The miR-499 is highlighted in yellow and the miR-208 is highlighted in red.
