# Supplementary material for: Evolution and genomic organization of muscle microRNAs in fish genomes
Source: BMC Evol Biol. 2014 Sep 25;14:196. doi: 10.1186/s12862-014-0196-x (PMC4177693; doi:10.1186/s12862-014-0196-x)

EVOLUTION AND GENOMIC ORGANIZATION OF MUSCLE MICRORNAS IN FISH GENOMES

Additional file 1: Multiple alignments of precursor sequences of the muscle-enriched miRNAs between vertebrate species and the secondary structure of consensus sequence. Light gray bar indicates mature sequence; Black gray bar indicates seed sequence.

Alignment of miR-1

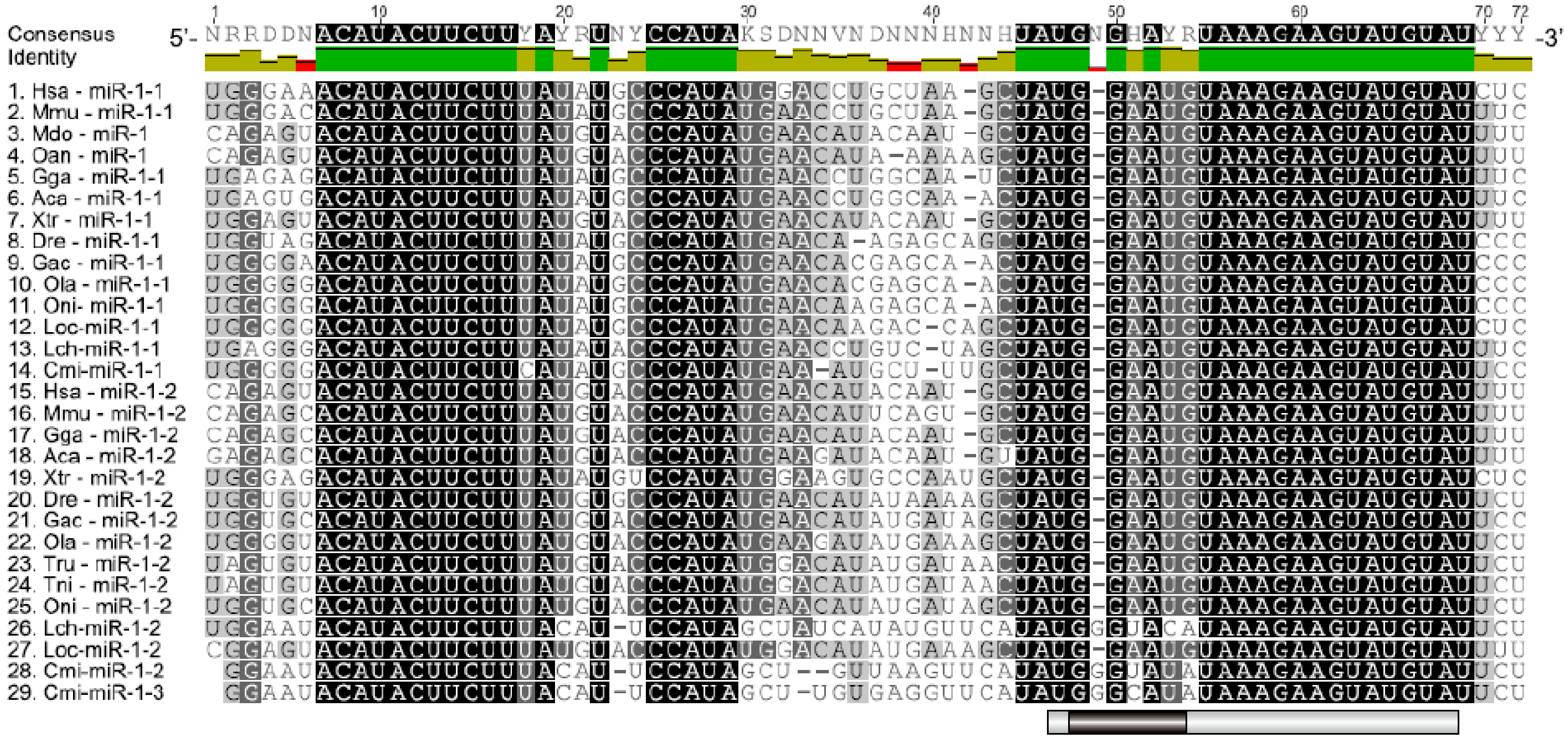

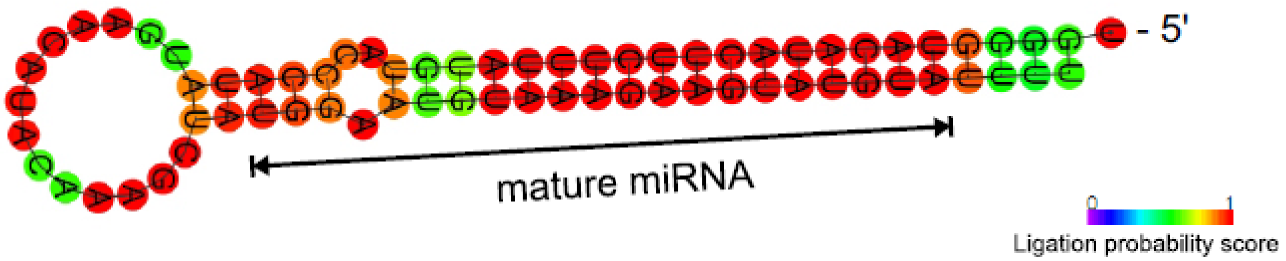

Alignment of miR-133a

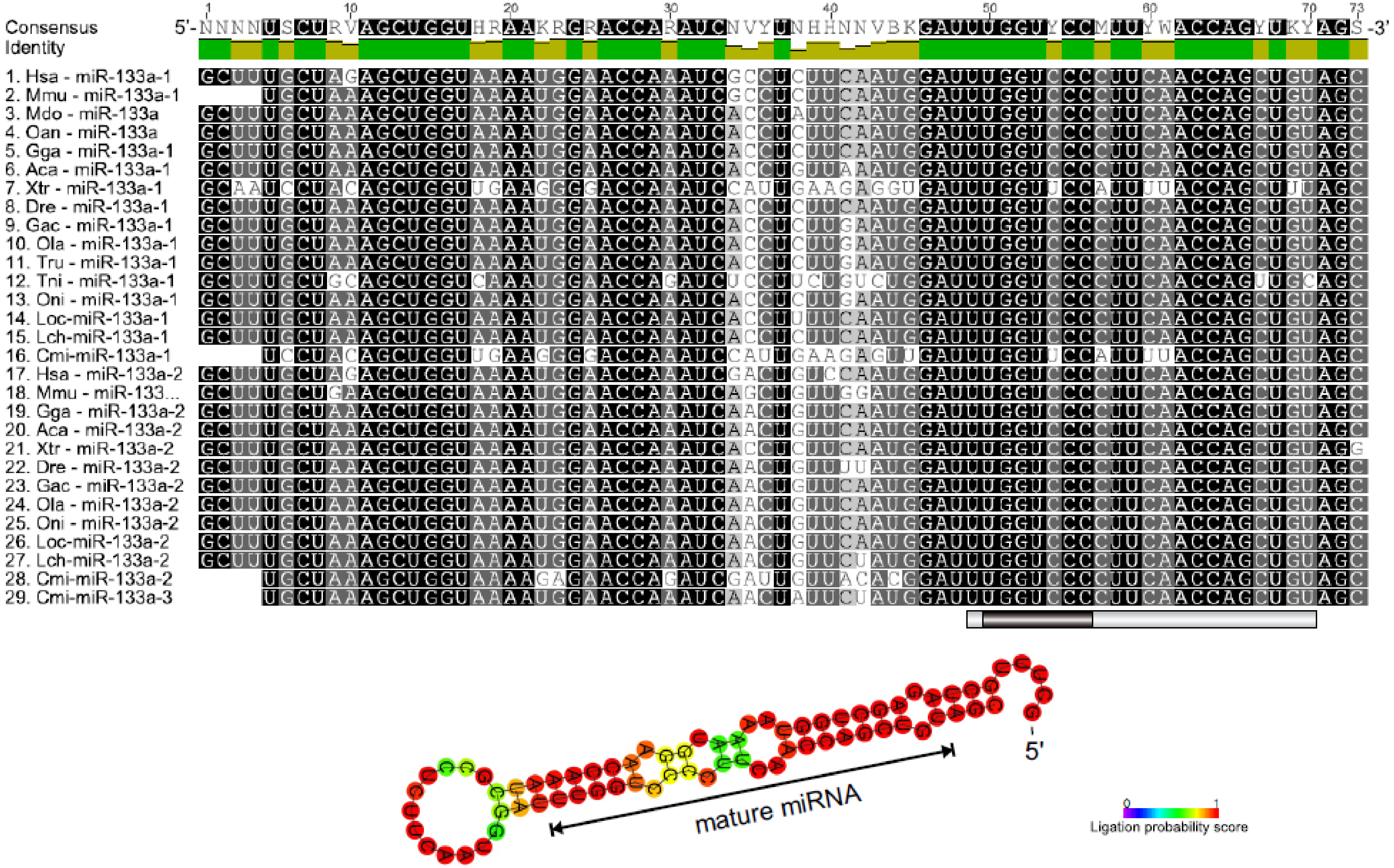

Alignment of miR-133b

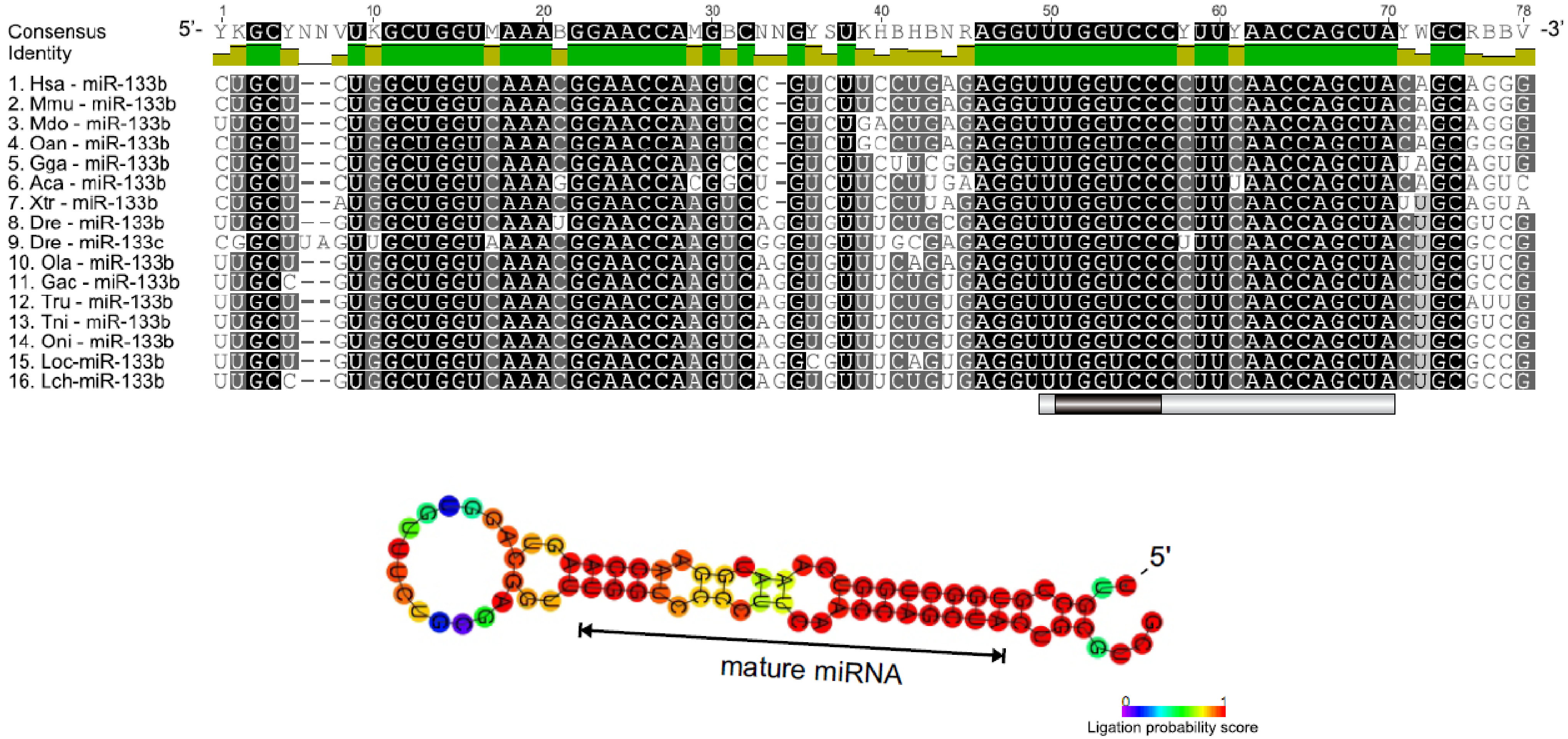

Alignment of miR-206

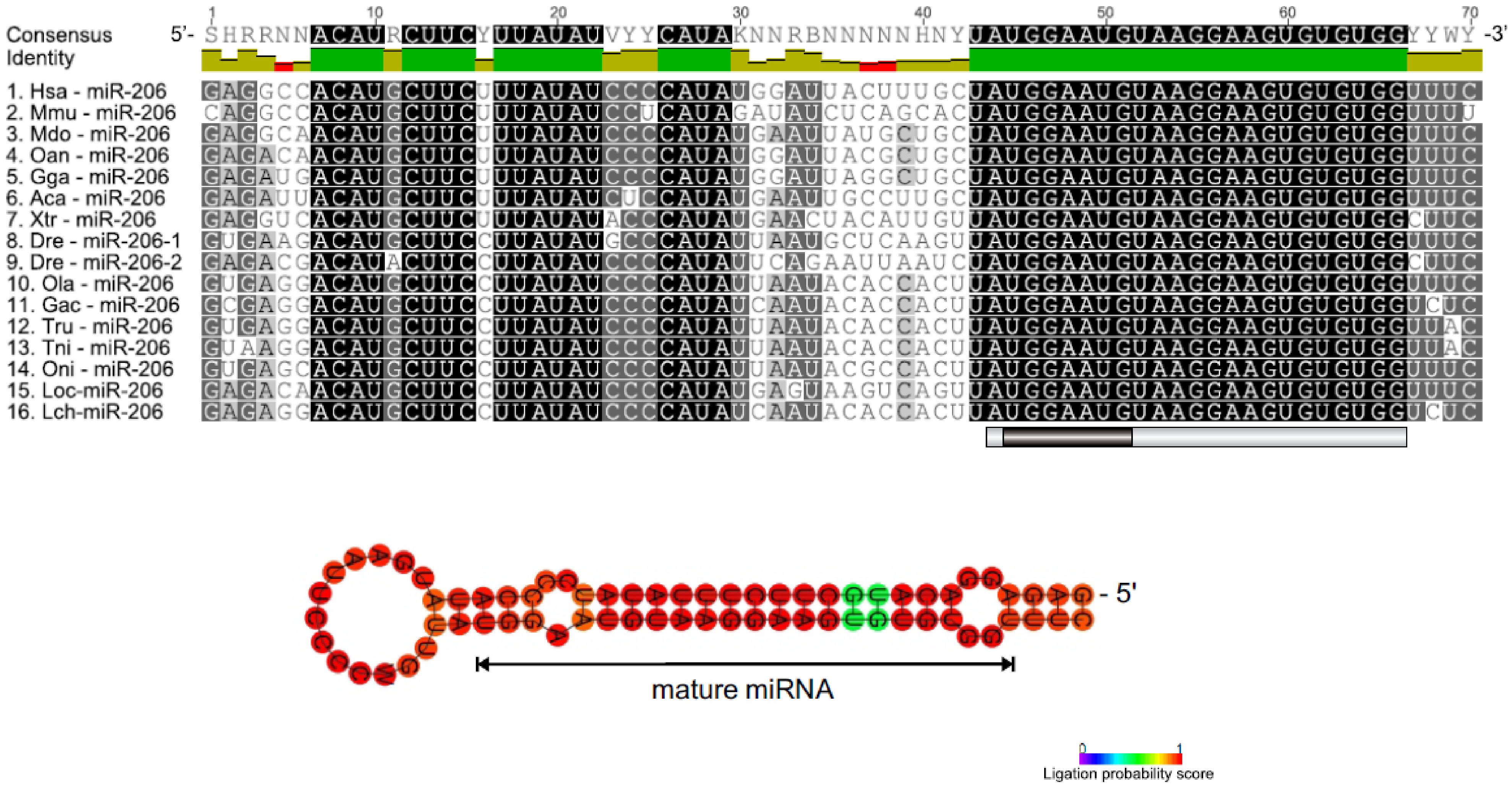

Alignment of miR-214

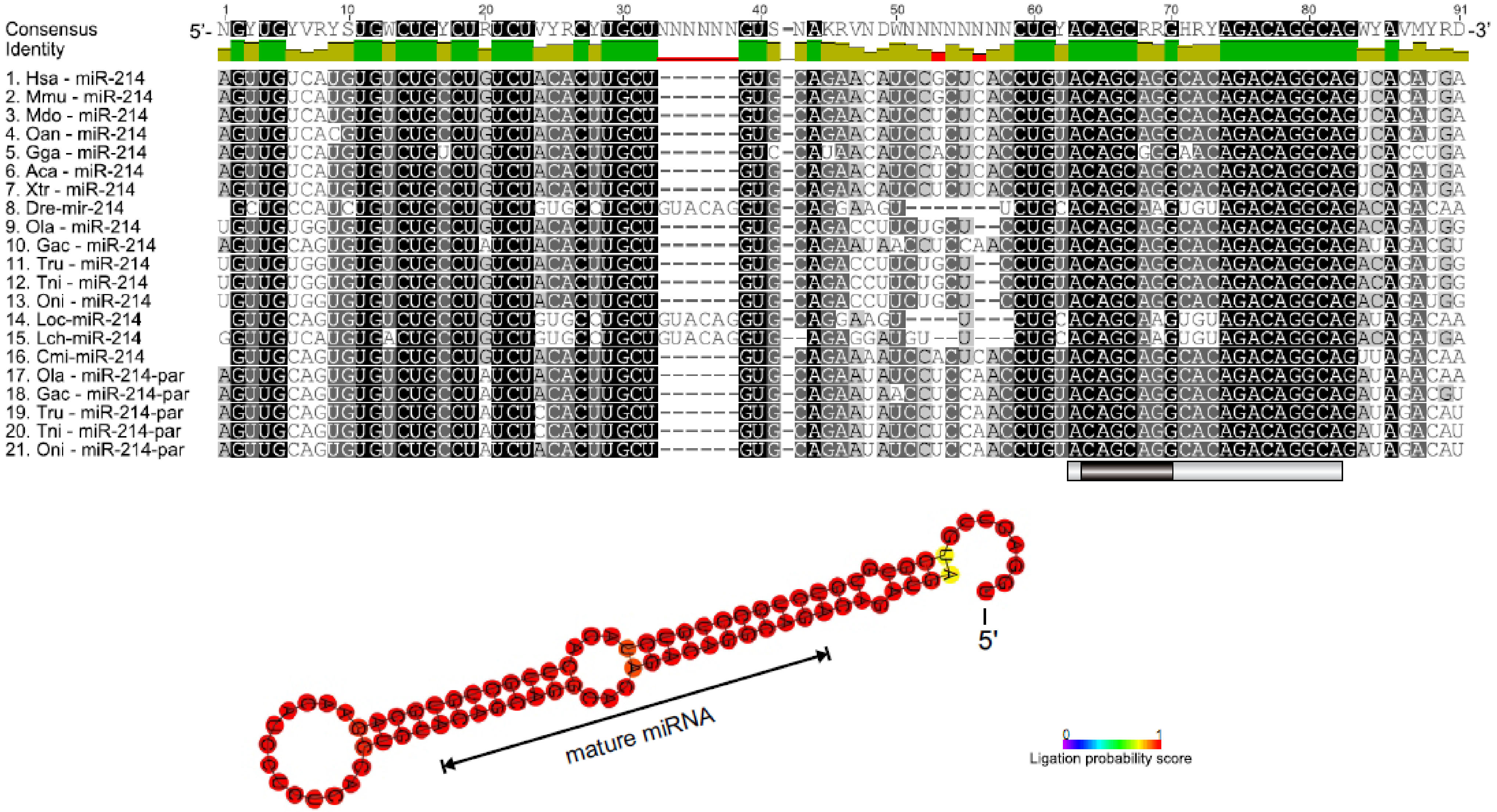

Alignment of miR-499

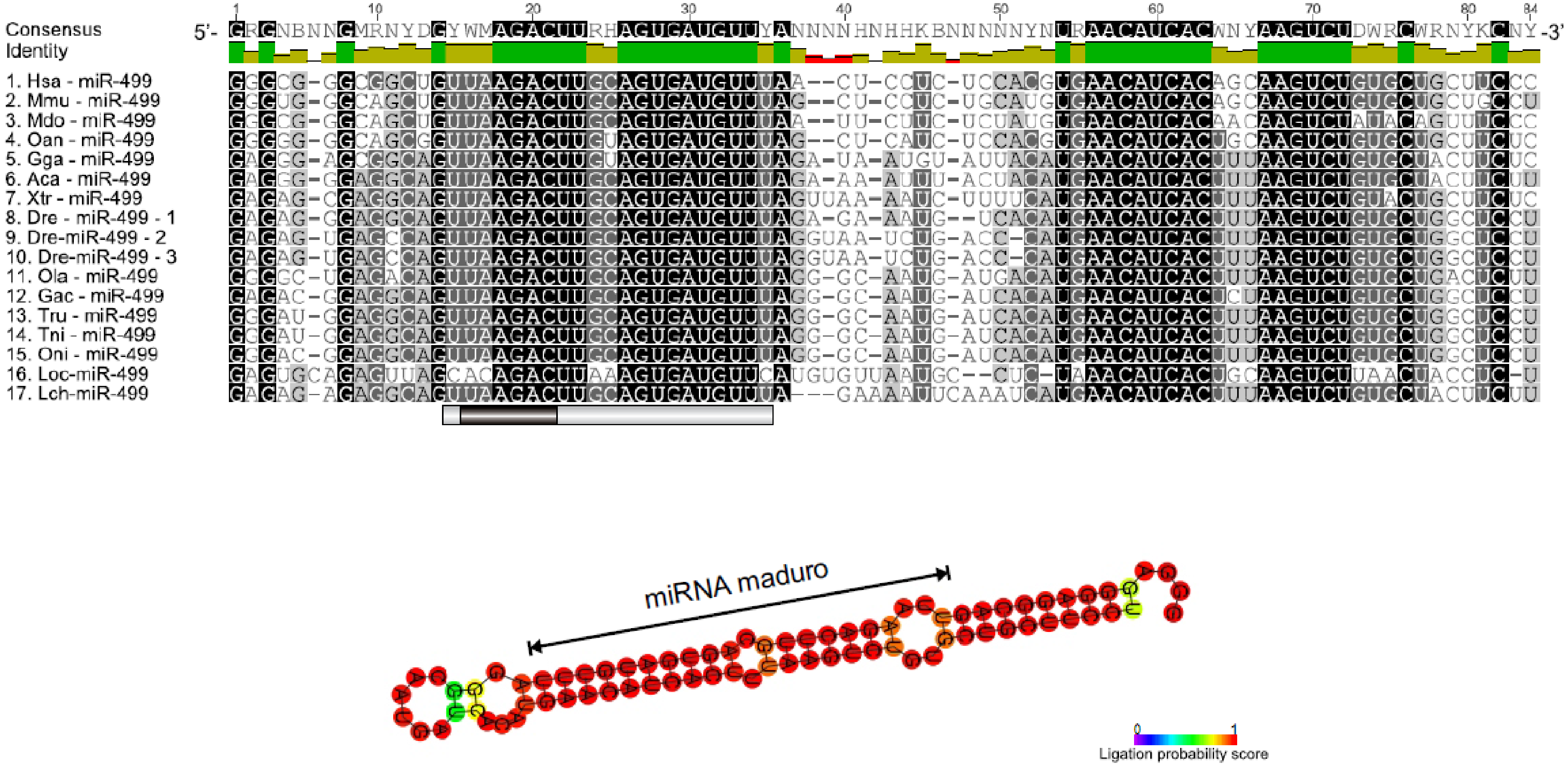

Supplement: Additional file 1: — Multiple alignments of precursor sequences of the muscle-enriched miRNAs between vertebrate species and the secondary structure of consensus sequence. Light gray bar indicates mature sequence; Black gray bar indicates seed sequence. [file 12862_2014_196_MOESM1_ESM.pdf]
