# Supplementary material for: Evolution and genomic organization of muscle microRNAs in fish genomes
Source: BMC Evol Biol. 2014 Sep 25;14:196. doi: 10.1186/s12862-014-0196-x (PMC4177693; doi:10.1186/s12862-014-0196-x)
Supplement: Additional file 2: Table S1. — Novel and formerly published data on muscle miRNAs in vertebrate genomes. [file 12862_2014_196_MOESM2_ESM.pdf]

EVOLUTION AND GENOMIC ORGANIZATION OF MUSCLE MICRORNAS IN FISH GENOMES

Additional file 2:

Table S1. Novel and formerly published data on muscle miRNAs in vertebrate genomes.

	Fishes									Amphibians	Reptiles	Birds	Mammals			
	Osteichthyes								Chondrichthyes				Monotremata	Marsupials	Eutherians	
**microRNAs**	Zebrafish (Dre)	Medaka (Ola)	Stickleback (Gac)	Tetraodon (Tni)	Fugu (Tru)	Nile tilapia (Oni)	Spotted gar (Loc)	Coelacanth (Lch)	Elephant shark (Cmi)	Frog (Xtr)	Lizard (Aca)	Finch (Tgu)	Ornithorhynchus (Oan)	Opossum (Mdo)	Mouse (Mmu)	Human (Hsa)
**miR-1-1**	O#	O#	O*	†*	†*	O*	O*	O*	O#	O*	O*	O*	O*	O*	O#	O#
**miR-1-2**	**O**#	**O**#	**O***	**O***	**O***	**O***	**O***	**O***	**O**#	**O***	**O***	**O***	**O***	**O***	**O**#	**O**#
**miR-133a-1**	**O**#	**O**#	**O***	**O***	**O***	**O***	**O***	**O***	**O**#	**O***	**O***	**O***	**O***	**O***	**O**#	**O**#
**miR-133a-2**	O#	O#	O*	†*	†*	O*	O*	O*	O#	O*	O*	O*	O*	O*	O#	O#
**miR-133b**	O#	O#	O*	O*	O*	O*	O*	O*	†*	O*	O*	O*	O*	O*	O#	O#
**miR-206**	O#	O#	O*	O*	O*	O*	O*	O*	†*	O*	O*	O*	O*	O*	O#	O#
**miR-208a**	†*	†	†*	†*	†*	†*	†*	**O***	†*	**O***	**O***	**O***	**O***	**O***	**O**#	**O**#
**miR-208b**	†*	†	†*	†*	†*	†*	†*	†*	†*	†*	†*	†*	**O***	**O***	**O**#	**O**#
**miR-214**	**O**#	**O***	**O***	**O***	**O***	**O***	**O***	**O***	**O***	**O***	**O***	**O***	**O***	**O***	**O***	**O***
**miR-214-par**	†*	**O***	**O***	**O***	**O***	**O***	†*	†*	†*	†*	†*	†*	†*	†*	†*	†*
**miR-499**	**O**#	O#	O#	**O**#	**O**#	**O**#	**O***	**O***	†*	**O***	**O***	**O**#	**O***	**O***	**O**#	**O**#

Note - † - miRNA undetected in the genome; O - Intergenic miRNA (blue O; shown as O); **O** - Intronic miRNA (red O; shown as bold **O**); * - data acquired in the present study; # - data previously published (Flynt et al., 2007; van Rooij et al., 2008; Bhuiyan et al., 2013; Tani et al., 2013); Dre – *Danio rerio*; Ola – *Oryzias latipes*; Gac - *Gasterosteus aculeatus*; Tni – *Tetraodon nigroviridis*; Tru – *Takifugu rubripes*; Oni – *Oreochromis niloticus*; Loc - *Lepisosteus oculatus*; Lch - *Latimeria chalumnae*; Cmi - *Callorhinchus milii*; Xtr – *Xenopus tropicalis*; Aca – *Anolis carolinensis*; Tgu – *Taeniopygia guttata*; Oan – *Ornithorhynchus anatinus*; Mdo – *Monodelphis domestica*; Mmu – *Mus musculus*; Hsa – *Homo sapiens*.
