# Supplementary material for: Evolution and genomic organization of muscle microRNAs in fish genomes
Source: BMC Evol Biol. 2014 Sep 25;14:196. doi: 10.1186/s12862-014-0196-x (PMC4177693; doi:10.1186/s12862-014-0196-x)
Supplement: Additional file 3: Table S1. — Structural characteristics of muscle miRNAs in fish genomes. [file 12862_2014_196_MOESM3_ESM.pdf]

EVOLUTION AND GENOMIC ORGANIZATION OF MUSCLE MICRORNAS IN FISH GENOMES

Additional file 3:

Table S1. Structural characteristics of muscle miRNAs in fish genomes.

miRNA	Zebrafish		Medaka		Stickleback		Tetraodon		Fugu		Nile tilapia		Spotted gar		Coelacanth		Elephant shark	
	Distance	Intron	Distance	Intron	Distance	Intron	Distance	Intron	Distance	Intron	Distance	Intron	Distance	Intron	Distance	Intron	Distance	Intron
**miR-1-1/-133a-2**	37.8kb	-	22.2kb	-	8.8kb	-	-	-	-	-	17kb	-	12.2kb	-	40kb	-	9.3kb	-
**miR-1-2/-133a-1**	2.1kb	12	2.7kb	12	3.1kb	12	2.5kb	12	2.5kb	12	3.4kb	12	3kb	11	3kb	12	4.6kb	11
**miR-206/-133b**	1.2kb	-	0.6kb	-	0.6kb	-	0.6kb	-	0.6kb	-	0.8kb	-	2kb	-	0.6kb	-	-	-
**miR-214**	-	10	-	15	-	15	-	13	-	13	-	14	-	14	-	13	-	13
**miR-214-par**	-	-	-	15	-	15	-	14	-	14	-	15	-	-	-	-	-	-
**miR-499**	-	18	-	-	-	-	-	19	-	19	-	17	-	18	-	18	-	-

Note - "Distance" is related to the approximately number of bases (kb) between miRNAs on the same cluster; "-" means unneeded information
